# Evaluation of health promotion in schools: a realistic evaluation approach using mixed methods

**DOI:** 10.1186/1471-2458-10-43

**Published:** 2010-01-28

**Authors:** Jeanine Pommier, Marie-Renée Guével, Didier Jourdan

**Affiliations:** 1 EHESP School of Public Health (Ecole des Hautes Etudes en Santé Publique), Av. du Professeur Léon Bernard, 35043 Rennes CEDEX, France; 2 Laboratoire PAEDI, School Health Education research unit and IUFM d'Auvergne (Auvergne Teacher Training College), Université Blaise Pascal, 36, avenue Jean Jaurès CS 20001, 63407 CHAMALIERES CEDEX, France

## Abstract

**Background:**

Schools are key settings for health promotion (HP) but the development of suitable approaches for evaluating HP in schools is still a major topic of discussion. This article presents a research protocol of a program developed to evaluate HP. After reviewing HP evaluation issues, the various possible approaches are analyzed and the importance of a realistic evaluation framework and a mixed methods (MM) design are demonstrated.

**Methods/Design:**

The design is based on a systemic approach to evaluation, taking into account the mechanisms, context and outcomes, as defined in realistic evaluation, adjusted to our own French context using an MM approach. The characteristics of the design are illustrated through the evaluation of a nationwide HP program in French primary schools designed to enhance children's social, emotional and physical health by improving teachers' HP practices and promoting a healthy school environment. An embedded MM design is used in which a qualitative data set plays a supportive, secondary role in a study based primarily on a different quantitative data set. The way the qualitative and quantitative approaches are combined through the entire evaluation framework is detailed.

**Discussion:**

This study is a contribution towards the development of suitable approaches for evaluating HP programs in schools. The systemic approach of the evaluation carried out in this research is appropriate since it takes account of the limitations of traditional evaluation approaches and considers suggestions made by the HP research community.

## Background

### Issues raised by evaluation in the field of health promotion

Schools are key settings for health promotion (HP). The contribution of HP to the health and well-being of pupils has been increasingly widely recognized [1-3]. However, the development of suitable approaches for evaluating HP in schools is still a major topic of discussion [2].

According to the definition given by the World Health Organization (WHO), evaluation aims to produce information that can be used by those who have an interest in the improvement and effectiveness of interventions [4]. However, evaluation in the field of HP has raised particular issues [5]. These issues are illustrated by Merzel and D'Afflitti (2003) who conducted a systemic literature review of 32 community-based HP programs. They identified five main issues: (1) methodological issues including the choice of the unit of assignment and analysis (individuals, communities, etc) and design and sampling issues; (2) the influence of secular trends and the difficulty of separating the impact of HP programs from these trends; (3) smaller-than-expected effects, i.e. relatively small effects are to be expected from community-level programs; (4) limitations of the HP programs including their duration, insufficient tailoring to reflect local conditions and the difficulty for community-level programs to ensure sufficient community penetration; and (5) limitations of theory because of the complexity of conceptualizing the relationship between multiple interventions and multiple levels of influence which makes it difficult to develop integrated explanatory theories as well as testable models [6]. Other authors [7,8] also pointed out further issues relating to the evaluation of HP: the complexity of the causality between an HP program and its effects, and the unsuitability of the experimental evaluation process for the HP values enshrined in the Ottawa Charter, i.e. the holistic nature of HP interventions and the values of participation, collaboration and empowerment.

Potvin et al (2008) identified three main challenges for those evaluating HP programs: (1) defining the activity to be evaluated in order to raise pertinent evaluation questions, (2) implementing an appropriate, rigorous research methodology, and (3) producing pertinent knowledge for actions [9]. In this context, the choice of methodology is of paramount importance.

This article presents a research protocol developing particularly the theoretical and methodological approach for evaluating HP interventions. It reviews the various evaluation approaches available and then describes the design developed and applied to the evaluation of an HP program in the specific context of the French school system.

### Evaluation approaches in the field of health promotion

Various evaluation approaches have been used in HP [8]. They are influenced by the multidisciplinary nature of HP and refer to various traditions. Within the positivist tradition, Rosen et al (2006) advocated the development of randomized designs that are appropriate and feasible for HP research [10]. Although for many decades randomized controlled experiments have dominated the impact assessment of social or health programs, there are many arguments that stress the artificiality of these approaches as well as the lack of useful information produced. It is usually not clear whether a program failed because it was built on poor conceptual foundations or it lacked a theoretical framework to identify causal mechanisms or because it was poorly implemented [8,11]. The WHO (1998) even concluded that "the use of randomized control trials to evaluate HP initiatives is, in most cases, inappropriate, misleading and unnecessarily expensive" (p.5 [4]).

Alternative approaches have been developed. Guba and Lincoln (1989) defined the fourth generation evaluation as "a form of evaluation in which the claims, concerns, and issues of stakeholders serve as organizational foci (the basis for determining what information is needed), that is implemented within the methodological precepts of the constructivist inquiry paradigm" (p.50, [12]). Over the past twenty years, other "participatory evaluation" approaches have been used increasingly frequently and various forms have been developed [13]. One of these forms is empowerment evaluation developed by Fetterman [14]. Wandersman (2005) defined empowerment evaluation as "an evaluation approach that aims to increase the likelihood that programs will achieve results by increasing the capacity of program stakeholders to plan, implement, and evaluate their own programs" (p.27, [14]).

Other authors [9,15] suggested using a realistic evaluation approach such as that developed by Pawson and Tilley [16]. Pawson and Tilley proposed studying the mechanisms that are triggered during the implementation of a program in a given context and establishing a relationship between the outcomes observed. Realistic evaluation aims to find out how a program works, for whom and under what circumstances. They considered a program to be a system of assumptions (i.e. action mechanisms leading to expected outcomes) that the evaluation process tests to develop a theory that can be applied and amended, for example, for the same program in different contexts. Thus, realistic evaluation considers the complexity of social programs and it may help to meet the challenges of evaluation in HP.

### The realistic evaluation framework

The realistic evaluation framework aims: (1) to understand the mechanisms through which HP interventions produces change; (2) to understand the contextual conditions necessary to trigger these mechanisms; and (3) to develop outcome pattern predictions according to the context and mechanisms triggered. These are the three guiding themes of the research strategy defined by Pawson and Tilley [16]. According to these authors, in a realistic evaluation approach, the outcomes of a HP program are explained by the action of specific mechanisms in specific contexts. It is thus essential in this type of evaluation approach to identify the mechanisms involved, i.e. what, within the program, produces change. The idea is to determine "which individuals, subgroups and locations might benefit most readily from the program, and which social and cultural resources are necessary to sustain the changes" (p.85, [16]). They name these configurations "context-mechanism-outcome pattern configurations" (CMO configurations). Realistic evaluators can then identify, modify, test and refine the CMO configurations. For these authors, a mechanism is "not a variable but an account of the make-up, behavior and interrelationships" of the processes which are responsible for the change, "a mechanism is thus a theory" (p.68, [16]). CMO configurations are developed on the basis of the literature on the subject being studied and on interviews with the stakeholders/participants of the program who play a key role in confirming, refuting or refining the theory.

The realistic evaluation framework does not require the use of a specific method. Indeed, Pawson and Tilley (1997) acknowledge that, when it comes to the choice of method, realistic evaluation can be based on methodological pluralism and thus on both qualitative and quantitative approaches.

According to Chen (1997), there are three types of configuration depending on different program evaluation contexts. In the first configuration, evaluation contexts require intensive information and have low availability of credible information and an open program system. In this type of configuration, it is more appropriate to use qualitative methods. In the second configuration, evaluation contexts require extensive, precise information, have high availability for credible information and a closed program system. This would require a quantitative approach. The third configuration concerns programs requiring information that is both intensive and extensive, that provides high access to some information but low access to other information and has the characteristics of both open and closed systems. In this case, the use of mixed methods is the most appropriate. Due to their complexity, HP interventions can be considered as an example of this last case.

### Mixed methods designs

Mixed methods (MM) and methodological pluralism are more and more often used within the HP field [7,8]. Using more than one method within a research project produces a more complete picture of the phenomena being studied [17]. Creswell and Plano Clark (2007) defined MM research as the combination of quantitative and qualitative approaches that provide a better understanding of research problems than either approach alone. The literature shows that MM research (1) provides strengths that offset the weaknesses of both quantitative and qualitative research; (2) provides more comprehensive evidence for studying a research problem than either quantitative or qualitative research alone; (3) helps answer questions that cannot be answered by qualitative or quantitative approaches alone; (4) encourages researchers to collaborate; (5) encourages the use of multiple worldviews or paradigms; (6) and is 'practical' in the sense that the researcher is free to use all possible methods to address a research problem [18].

The MM approach can have different designs depending on how qualitative and quantitative approaches are combined. Creswell and Plano Clark (2007) classified the MM designs into four major types:

(1) triangulation: its purpose is to obtain different but complementary data on the same topic to best understand the research problem;

(2) embedded: one data set provides a supportive, secondary role in a study based primarily on the other data type;

(3) explanatory: a two-phase MM design where qualitative data helps to explain or build upon initial quantitative results;

(4) exploratory: the results of the first method (qualitative) help to develop or form the basis of the second method (quantitative).

Creswell and Plano Clark (2007) identified three factors to which the choice of a research design is related: the timing of the use of the collected data, the relative weight of the quantitative and qualitative approaches and the approach to mixing the datasets.

## Methods/Design

This section covers research questions, the HP program, the sample, data collection and data analysis. It takes account of the guidelines for reporting observational epidemiological data given in the STROBE initiative [19]. The study was conducted in France and was designed to evaluate an HP program implemented in primary schools.

### Research questions

In the literature concerning research methodology, the purpose of a research study is defined as the reason or reasons for carrying out the study. These purposes are interrelated with the research questions and methods. Newman et al [20] stress the importance for researchers to clarify their thinking about the purpose of their studies. They developed a typology of research purposes as a conceptual tool. They defined nine general goals for social science research studies: predict, add to the knowledge base, have a personal, social, institutional and/or organizational impact, measure change, understand complex phenomena, test or generate ideas, inform and examine the past.

According to Newman's typology [20], this research project aims to add to the knowledge base of HP in schools. However, since it is a complex research project, this general purpose can be further refined and the overall project can be divided into two stages. The first stage, based on an "inductive theoretical drive" [17], explores the individual and collective HP practices of French teachers and studies the mechanisms used to implement HP programs. The second stage, based on a "deductive theoretical drive" [17], focuses on measuring changes arising from the HP program among children, teachers, families and school communities.

Based on these hypotheses and research purposes, the research questions can be defined as follows:

- What are the mechanisms and contextual factors that allow the school community to develop an HP approach?

- How do the strategies developed through the program influence the development of teachers' HP practices and the schools' health promoting environment? How do these practices affect well-being in the schools? What is the influence of the program on the children's perceived life skills?

### The health promotion program

**Figure 1:**
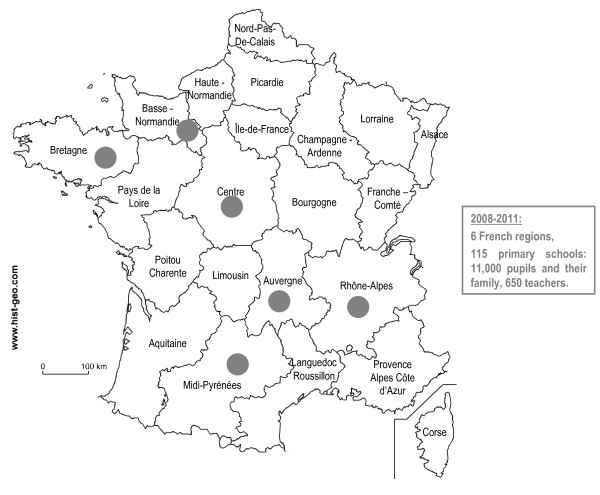
**French regions involved in the project**.

The French system is national and centralized. Schools set a low priority on HP [21]. Professionals in the workplace are not always aware of their HP role [22]. An HP program was designed specifically for this context to address these issues and enable the school staff to implement an HP policy [23]. A four-year pilot study (2003-2007) was carried out in 21 schools [23,24]. During this pilot stage, there were in-depth interviews with the program designers and those involved locally, observations were made and documents were collected. Following this pilot stage, a wider program was designed and implemented in 115 schools in 6 French regions. The project started in 2008 and will continue until 2011 (table 1 and figure 1).

**Table 1:** Main features of the health promotion program to be evaluated

Objectives of the program	- to promote children's social, emotional and physical health by contributing to children's well-being at school [44,45] and enhancing their life skills [28,36]; - to develop relevant HP teaching practices and the health promoting environment in schools, - to develop sustainable HP projects in schools by the empowerment of local stakeholders.
**Theoretical background**	The program takes into account the most recent international publications and data concerning the development of school HP programs [2,8,43,46]. This implies the development of a progressive sustainable program:
	- taking into account the development of the children,
	- linking health to educational issues as well as integrating them into ongoing school activities,
	- communication with parents and communities,
	- training and support of school professionals and accessibility of resources and other methodological tools.
	It also takes into account the special features of the French system. The program is a combination of top-down and bottom-up approaches and therefore the characteristics of the actions implemented in each school may vary [47].

**Implementation**	The program is being implemented in 115 schools in 6 French regions. The program started in 2008 and will continue until 2011. In each region, a support team is in charge of the implementation of the HP program. These support teams were trained to provide training and support to the teachers and the schools concerning the HP program, its principles, values, resources and evaluation. Pedagogical resources are provided for each school. Prior to this program, a four-year pilot study was carried out in 21 schools [23,24]. An ethics committee has also been set up.

HP: health promotion

The evaluation framework for this HP project was based on the "theory-driven" approach to evaluation defined by Chen and Rossi (1983). This approach "is not the global conceptual scheme of the grand theorists, but more prosaic theories that are concerned with how human organizations work and how social problems are generated [...]. What we are strongly advocating is the necessity for theorizing, for constructing plausible and defensible models of how programs can be expected to work before evaluating them" (p.285, [11]). For the authors, this implies identifying theory consistent with social science knowledge and theory.

**Figure 2:**
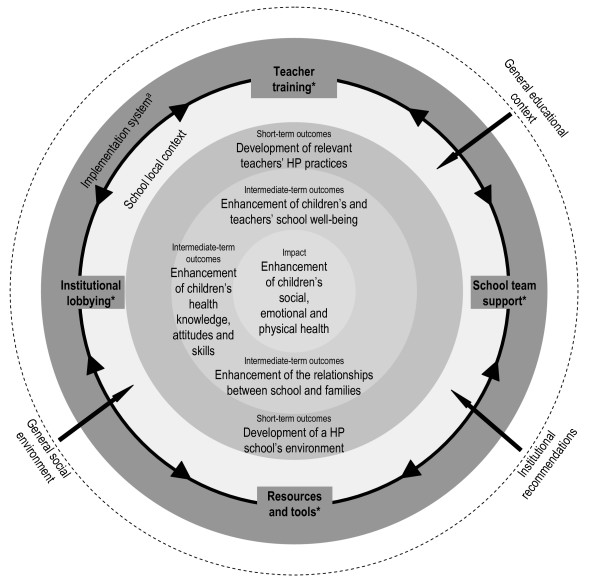
**Theory-of-change model of a health promotion program in school setting**. a Implementation system - an intervention once enacted must be carried out through an implementation system that includes rules, organizational structures and personnel who have been given the responsibility to administrate the intervention (Chen & Rossi, 1983). * Strategies. HP: heath promoting.

Figure 2 presents the theory-of-change model underlying this HP program [25]. It suggests that the strategies developed through the program (teacher training, school team support, resources and tools, and institutional lobbying) can positively influence teachers' HP practices [26] and the schools' health promoting environment and enhance the well-being of children and teachers at school, improve the relationship between schools and families [1,27], develop children's health knowledge, attitudes and skills [28] and possibly improve children's social, emotional and physical health [28]. This model is based on the assumption that the outcomes and strategies interact with the general and local contextual factors and the way in which the program is implemented (i.e. rules, organizational structure and personnel who are responsible for managing the program) [11].

Many factors govern the ways in which schools can develop and implement HP programs: sustainable commitment on the part of institutions and communities, a favorable environment, such as the support of the school head, and factors linked to the implementation of the program itself. A program cannot be implemented using a top down approach without consulting those involved locally and without taking into account of the local situation. The HP program was proposed in 2007 to all 31 French teacher training institutes. These institutions have the authority and legitimacy to sustain a school HP program. Ten institutes in 10 different French regions agreed to participate in the project. Six regions were able to gain institutional support and set up a support team to implement the program and collect data. Within those 6 regions, a total of 115 schools were given institutional support to participate to the program (figure 1).

### Evaluation protocol

In the evaluation of this HP program, the mechanisms triggered by the program are described with reference to the literature and the results of the pilot study [24]. Each school involved in the project is considered as a separate unit in a specific context. The mechanisms triggered are determined together with the way in which they produce the outcomes in each of these specific contexts. This leads to the definition of a theory detailing which mechanisms of the program work in which context to produce which outcomes and for whom. As discussed in the previous section, an MM approach is appropriate for realistic evaluation. Some authors may argue that using both quantitative and qualitative methods creates tensions since they are based on contrasting assumptions about social phenomena. However, this evaluation is more concerned with providing an overall understanding of the nature of the theory-of-change model and how it actually operates than with methodological purity. However, the qualitative and quantitative approaches meet the standards of rigor for both methods even though the integrated design requires structural changes in the methods themselves that may become harder to meet [29]. A quantitative and qualitative approach is, therefore, required to explore the research questions and deal simultaneously with the inductive and deductive theoretical drives.

**Figure 3:**
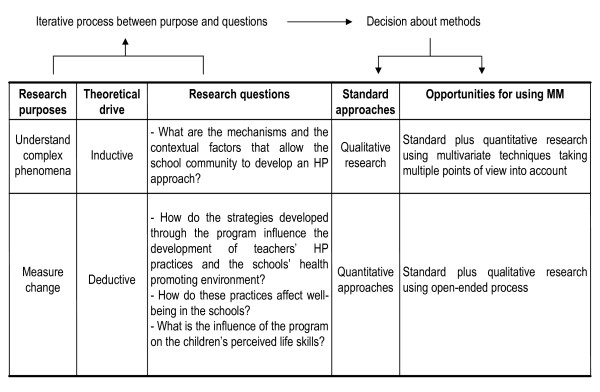
**Iterative process from research purposes to opportunities to use mixed methods**. HP: health promotion, MM: mixed methods.

Figure 3 presents a synthesis, as proposed by Newman [20], to explain the construction of the research from research purposes to the choice of MM approaches. It details the research purposes, the theoretical drives, the research questions and the methods.

**Figure 4:**
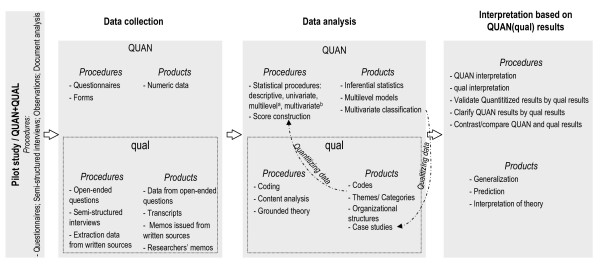
**Mixed methods embedded design of the research: data collection, analysis and interpretation procedures and products**. QUAN: quantitative, qual: qualitative. a: regression (logistic, linear...). b: principle component analysis, multiple correspondent analysis, classification.

**Figure 5:**
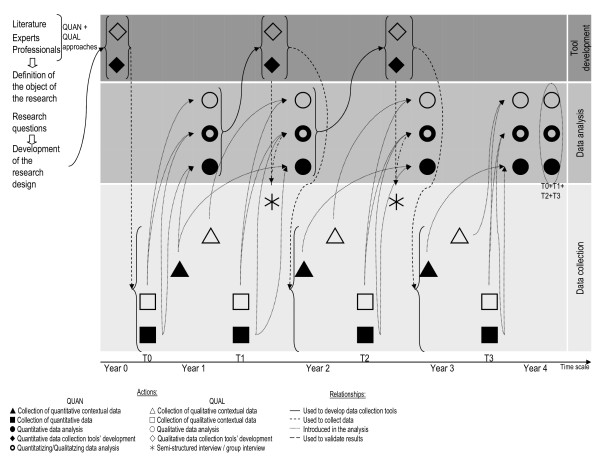
**Tool development, data collection and analysis: relationships and iterative process between qualitative and quantitative approaches**.

According to the factors that influence the choice of an MM design as defined by Creswell and Plano Clark [18], this research project is based on an embedded design: QUAN(qual). The research questions focus on quantitative data to measure changes and qualitative data plays a secondary supportive role in exploring HP practices. Data is collected concurrently: quantitative numerical data is collected from questionnaires and forms and qualitative data (text data, transcripts and memos) from open-ended questions included in questionnaires and forms and from semi-directed interviews. The data is analyzed using standard quantitative and qualitative analysis, quantitization and qualitization [29]. Quantitative variables are presented in table 2, and table 3 presents the categories of general mechanisms and contextual factors that may play a role in the desired outcomes. The interpretation is quantitative, qualitative and combined where the quantitative results are clarified by the qualitative results, in order to generalize the findings, predict and interpret theory. Figure 4 presents the MM embedded design of this research project and summarizes the data collection and analysis procedures and products as well as the QUAN(qual) interpretation stage. In this research, qualitative and quantitative methods are mixed throughout all phases of the project from the design stage through data collection to data interpretation. Figure 5 presents the mixing process and describes the relationships and iterative process between the qualitative and quantitative approaches, the different datasets and the project phases.

**Table 2:** Deductive research questions and variables' description

Research question		Dependent variables		Independent variables	
		Content	Description	Content	Description
What is the influence of the strategies developed through the intervention in the development of HP practices at school and class levels?			- qual:	Perception of HP	- qual
		Collective HP practices	yes/no - qual: types	Institutional recommendations/policy	- qual
			of practices	Solicitations	- qual
				Perception of HP	- qual

				Interest	- qual
				Perceived self-efficacy	- qual
			- qual:	Motivation	- qual
		Individual HP practices	yes/no - qual: types	Institutional recommendations/policy	- qual
			of practices	Availability of pedagogical tools	- qual
				Training	- qual + quan
				Community's solicitations	- qual
				School climate	- quan: score
				School characteristics	- qual

What is the influence of These practices on well-being in the school setting? On the relationship established with parents?	From teachers' point of view	Perceived school climate (teachers)	- quan: score	Collective HP practices	- qual: yes/no
			
	From children's point of view	Perceived school climate (children)	- quan: score	Individual HP practices	- qual: types of practices
		Violence	- quan: score	School characteristics (rural/urban; educational priority status; school size; socio-economical status...)	- qual
			
	From families' point of view	Perceived school climate (families)	- quan: score		
		Relationship with parents	quan: score	The actors' perceived school climate	- quan: score
				As above with the addition of:	

What is the impact of the perception of the life in school on children's perception of their life skills?		Children's perception of their life skills	- quan: score	Violence	- quan: score
			- qual	Relationship with parents	- quan: score

quan: quantitative variable, qual: qualitative variable, HP: health promotion

**Table 3:** Categories of general mechanisms and contextual factors that may play a role in the desired outcomes

Mechanisms	Contextual factors
Outcome 1: Development of an HP approach at the school level	

- Development of collective work skills - Integration of HP in the school's project - Common perception of HP - Presence of a leader	- National institutional will - Local institutional support - Training means and trained resources - Availability of resources - Community involvement

Outcome 2: Development of teachers' HP practices	

- Development of personal skills - Perception of HP - Perceived self-efficacy - Capacity to use resources - Capacity to integrate HP considerations in their practices - Motivation and interest - Teachers' empowerment	- Local institutional support - HP integrated in the school's project - Training means and trained resources - Availability of resources - Existence of an HP approach within the school - Perceived needs of children

Outcome 3: Development of children's school well-being	

- Health education activities - Involvement of children in HP project - Development of personal life skills	- Development of a global HP school approach integrating parents and wider community - Teachers having HP practices - Development a supportive psychosocial and physical environment

HP: health promotion

These items are based on the literature and the personal experience of the researchers (for example, from the pilot study). The mechanisms and contextual factors vary within each school.

### Sample

As one of the central outcomes of the study is the teachers' HP practices, our sample size was defined based on this outcome. Previous French studies showed that the prevalence of teachers' HP practices is at least 70% [30]. The calculation of the minimum sample size with an acceptable error of 5% and a margin of error of 5% using the Cochran's formula [31] gives the results of 314 individuals. This result provides us a rough guideline of our minimal sample size [32]. If we take into consideration a response rate of 65%, our sample size should be of 483. In this study, 650 teachers are concerned by the intervention ensuring the desired statistical power and precision. This research aims to produce inferences for the teachers' national population. Therefore frequency weights [33] will be used according to three criteria: teachers' location (rural vs. urban), size of the school where teachers work (small < 4 classes and big > or = 4 classes) and socioeconomic status of the school area where teachers work (privileged vs. underprivileged).

Concerning the children, only those who were able to answer to a 30 minutes self-administrated questionnaire were concerned by the evaluation procedure. This decision was taken based on the results of the pilot study. This corresponds to 3^rd ^to 5^th ^grade students. This represents 4,690 pupils of the participating schools. Parents consent was required.

All of the families of the participating schools were invited to participate which represents approximately 8,000 families.

All the schools participating in the program were concerned by the evaluation procedure, i.e. 115 schools.

### Data collection

The variables of interest were defined on the basis of the research questions and the theory-of-change of the HP program. Table 2 shows the dependent variables linked to the deductive research questions and the independent variables. Table 3 presents the mechanisms and contextual factors identified in the literature and in the results of the pilot study. Data collection tools (four questionnaires and two forms to be completed by semi-directed interview) were developed from this general canvas and the work done in the pilot stage. The questionnaires were registered at a national ethics committee, the "Commission nationale de l'informatique et des libertés" (CNIL), the national board in charge of data protection and liberties http://www.cnil.fr/english/the-cnil/.

#### Data collection tools

Four questionnaires were drawn up for children, teachers, parents and school communities. Two forms were designed to collect contextual information.

#### ▪ Children's questionnaire

This questionnaire was designed to collect data on children's perception of their life in school and life skills. Children's perception of their life in school was studied through questions on the school climate and on their perception of their relationship with other children, teachers and adults working in the school. This part of the questionnaire was based on the questionnaire developed by Debarbieux at the European observatory of school violence [34,35] which was adapted and used in the pilot study. The second part of the questionnaire on children's perception of their life skills was based on the WHO definition [36], Five basic areas of life skills were identified: (1) decision-making and problem-solving, (2) creative thinking and critical thinking, (3) communication and interpersonal skills, (4) self-awareness and empathy, and (5) coping with emotions and coping with stress. Particular attention was paid to the presentation of the questionnaire and face scales [37] were used whenever possible.

#### ▪ Teachers' questionnaire

This questionnaire was designed to collect data on teachers' attitudes to HP, on their own practices and factors that might influence them (facilitators, barriers, etc), on their motivation, interest in HP and feeling of competency in HP, as well as on their perception of the life in their school (school climate, violence, etc) This questionnaire was primarily developed in 1991 in a study on teachers' practices and attitudes to HP [22], it was amended and used in the pilot study.

#### ▪ Families' questionnaire

This one page questionnaire was designed to collect data on how the families perceived life at the school, their relationships with the school and their involvement in the school's activities [34,35].

#### ▪ School communities' questionnaire

This questionnaire was designed to collect data on the local community's attitudes to HP, on HP activities implemented in schools and on how schools can be considered an HP environment. The last part of the questionnaire was based on the criteria defined by the IUHPE in 2005 and reviewed in 2008 [38]. These defined six essential components of an HP school: (1) the development of health school policies, (2) the attention given to the school's physical environment, (3) the attention given to the school's social environment, (4) the development of individual health skills and action competencies, (5) the development of community links, and (6) the links with health services. The first part of the questionnaire was based on the qualitative work in the pilot study [24].

#### ▪ Forms

Two forms were drawn up to collect contextual and process data, one on the school's structural and social background and one on the implementation of the program in each region.

#### ▪ Semi-directed interviews

Two main interview guides were drawn up: one for the school community (teachers, parents, and children) and one for the support teams. Members of each support team are interviewed to find out their perception of the implementation of the program: facilitators, obstacles, results observed and added-value of the program. The second interview guide is adapted to each group, the main common themes being perception of the program, facilitators, obstacles and results observed.

#### Procedure for validating the data collection tools

Data collection tools must be validated to ensure the rigor and quality of the research. All the tools were first tested for face and content validity by consulting the research team who undertook the pilot study, the scientific committee for the project and the support teams in the regions. The questionnaires were validated by structured interviews with 10 persons from each group (teachers, children and parents). The reliability of the teachers' and children's questionnaires was then tested. The questionnaires were administered in real conditions to a sample of 30 individuals from each target group. The data was entered and tested for internal reliability using the Cronbach alpha coefficient method whenever appropriate (teachers' questionnaires: 0.80 and 0.81 respectively for the questions on teachers' conceptions and teachers' perceived self-efficacy; children's questionnaire: 0.62 and 0.66 respectively for the questions on perceived school climate and perceived violence). The questionnaires were administered a second time to the same sample 15 days later. The new data was entered and analyzed to check the reliability using the Kappa coefficient method whenever appropriate. At the end of each stage of the validation procedure, the tools were modified according to the conclusions drawn.

#### Data collection procedure

Data is collected from pupils, parents, teachers, school communities and support teams in a three year multiple time series design, at the beginning of the project and at the end of each school year (T_0_, T_1_, T_2_, T_3_). The questionnaires are self-administrated and distributed by the members of the support team.

The results of each questionnaire are returned to the schools once a year. A specific user-friendly document was created and validated by those involved and the results are communicated to school communities by the support team. A discussion will be organized with those involved to collect feedback on their perception and the conclusions drawn.

Interviews with the support teams are conducted once a year. Moreover, in each region, a sample of schools is selected according to their involvement in the program (from low to high). Teachers, school staff and some parents and children are interviewed. All the interviews are recorded with the approval of the participants and transcribed.

### Data analysis

Quantitative analysis (descriptive, univariate, multivariate and multilevel) is performed using R 2.2.1 http://cran.r-project.org/, Stata http://www.stata.com/ and SAS http://www.sas.com/ software. NVivo http://www.qsrinternational.com/ software is used to analyze the qualitative data and a content analysis is carried out [39]. Furthermore, some qualitative data is quantitized [29] into quantitative data and statistically analyzed. Some of the results of the quantitative analysis are qualitized [29] to mount a grounded theory process [40]. The analytical framework is presented in tables 2 and 3. The analysis procedures that will be used are presented in figure 4.

## Discussion

This article presents the theoretical and methodological approach of research designed to evaluate an HP program in French schools. It presents the HP evaluation issues and describes the main evaluation approaches used in the field and gives a detailed illustration of the evaluation framework applied to an HP program in French schools. It discusses how the realistic evaluation of such an HP program is a valuable approach to take account of the general and local context. The systemic approach of the evaluation carried out in this project is appropriate since it takes into account the limits of traditional evaluation approaches and considers the suggestions of the HP research community [8,9,41,42]. Furthermore, the evaluation design used as an illustration in this article is a structured utilization of MM. It describes in particular the interactions between the qualitative and quantitative approaches and the added value of each approach. In his review of the evidence on school HP, Stewart-Brown (2006) stated "It is becoming increasingly clear that research on promoting health requires a variety of methodological approaches, including process- and outcome-based evaluation, and quantitative and qualitative methods." [43]. This article is a contribution towards the improvement of suitable approaches for evaluating HP programs in schools. It is not possible to give a definitive answer: the pros and cons of different research methodologies are still being discussed. On the basis of this analysis of the available frameworks, it is clear that the realistic evaluation approach could enable researchers to find an appropriate solution for evaluating comprehensive HP programs in schools. Developing research programs based on this framework could help to bridge the gaps in showing the effectiveness of school HP.

## Competing interests

The authors declare that they have no competing interests.

## Authors' contributions

This article was drafted by JP and MRG and the manuscript was revised by DJ. The project design was developed by DJ and JP. All authors were involved in implementing the project and in developing the evaluation design. All authors read and approved the final manuscript.

## Pre-publication history

The pre-publication history for this paper can be accessed here:

http://www.biomedcentral.com/1471-2458/10/43/prepub
